# Assessing Diet Quality of Indigenous Food Systems in Three Geographically Distinct Solomon Islands Sites (Melanesia, Pacific Islands)

**DOI:** 10.3390/nu13010030

**Published:** 2020-12-23

**Authors:** Chris Vogliano, Jessica E. Raneri, Josephine Maelaua, Jane Coad, Carol Wham, Barbara Burlingame

**Affiliations:** 1 School of Health Sciences, Massey University, Wellington Campus, Wellington 6021, New Zealand; barbara.burlingame@gmail.com; 2 Department of Food Technology, Safety and Health, Ghent University, 9000 Ghent, Belgium; jessicaraneri@gmail.com; 3 Faculty of Nursing, Medicine and Health Sciences, Solomon Islands National University (SINU), Honiara P.O. Box R113, Solomon Islands; josephine.maelaua@sinu.edu.sb; 4 School of Food and Advanced Technology, Massey University, Manawatū Campus, Manawatū 4442, New Zealand; j.coad@massey.ac.nz; 5 School of Sport, Exercise and Nutrition, Massey University, Albany Campus, Auckland 0632, New Zealand; c.wham@massey.ac.nz

**Keywords:** indigenous peoples, food systems, sustainable diets, wild foods, food security, nutrition, SDG 2, Pacific Islands, biodiversity

## Abstract

Indigenous Solomon Islanders, like many living in Pacific Small Island Developing States (PSIDS), are currently experiencing the global syndemic—the combined threat of obesity, undernutrition, and climate change. This mixed-method study aimed to assess nutrition transitions and diet quality by comparing three geographically unique rural and urban indigenous Solomon Islands populations. Participants in rural areas sourced more energy from wild and cultivated foods; consumed a wider diversity of foods; were more likely to meet WHO recommendations of >400 g of non-starchy fruits and vegetables daily; were more physically active; and had significantly lower body fat, waist circumference, and body mass index (BMI) when compared to urban populations. Urban populations were found to have a reduced ability to self-cultivate agri-food products or collect wild foods, and therefore consumed more ultra-processed foods (classified as NOVA 4) and takeout foods, and overall had less diverse diets compared to rural populations. Clear opportunities to leverage traditional knowledge and improve the cultivation and consumption of underutilized species can assist in building more sustainable and resilient food systems while ensuring that indigenous knowledge and cultural preferences are respected.

## 1. Introduction

Indigenous Solomon Islanders, like many living in Pacific Small Island Developing States (PSIDS), are currently experiencing the global syndemic, which is the combined threat of obesity, undernutrition, and climate change [1]. Climate change is predicted to challenge accumulated traditional food knowledge through changing landscapes and weather patterns and rising sea levels, with the highest sea rises predicted to occur near the equator [2]. The Solomon Islands (Melanesia) are considered to be a biodiversity hotspot, containing vast genetic diversity and traditional knowledge, which are valuable assets towards ensuring resilient and sustainable food systems in the future [3,4,5]. However country-level import data suggest that ultra-processed foods such as carbonated soft drinks, baked foods, processed meats, noodles, and sweet biscuits make up the majority of imports in transitioning PSIDS [6]. Additionally, mounting evidence suggests that sharp rises in tropical log exports are degrading local fisheries [7], being linked to decreased ecological resilience and a rise in wealth inequality among Solomon Islanders [8]. Therefore, this study aimed to assess diet quality and food system transitions across three geographically unique Solomon Islands populations.

According to the Committee on World Food Security, food security and nutrition policy are best approached within a sustainable food system framework underpinned by the right to food [9]. A sustainable food system has been defined as “a food system that delivers food security and nutrition for all in such a way that the economic, social and environmental bases to generate food security and nutrition for future generations are not compromised” [10]. The majority of Solomon Islanders live in rural communities and have traditionally relied on subsistence agriculture and fish as their primary source of nutrition [11]. However, knowledge of recent nutrition transitions and associated nutrient contributions of indigenous food systems is limited, particularly when comparing rural populations with rapidly urbanizing populations.

One recent dietary diversity study conducted in the peri-urban population of Malaita [12] found that diet diversity is higher in rural areas compared with peri-urban populations, but it is unknown as to how these findings relate to the urban capital of Honiara, where most urban Solomon Islanders reside. Another recent study found poor dietary diversity among women and young children in rural Solomon Island populations (Western Province and Malaita), with diets dominated by grains, white roots, tubers, and plantains [13]. To understand the relationship between traditional knowledge and barriers to healthy diets, it is critically important to assess diet quality; diversity; sourcing patterns; and the knowledge, attitudes, and practices of those living in geographically unique areas in Solomon Islands [12].

This study aimed to assess nutrition transitions by comparing quantitative anthropometric measurements, diet quality measurements, and food sourcing patterns among three geographically unique rural and urban indigenous Solomon Island populations. Additionally, qualitative key informant interviews were used to contextualize the quantitative data by identifying emerging concerns towards healthy and sustainable foods systems. Quantitative data were collected through the administration of a comprehensive nutrition survey and participatory quantitative interviews with village elders and other key informants

## 2. Methods

### 2.1. Study Sites

**Figure 1:**
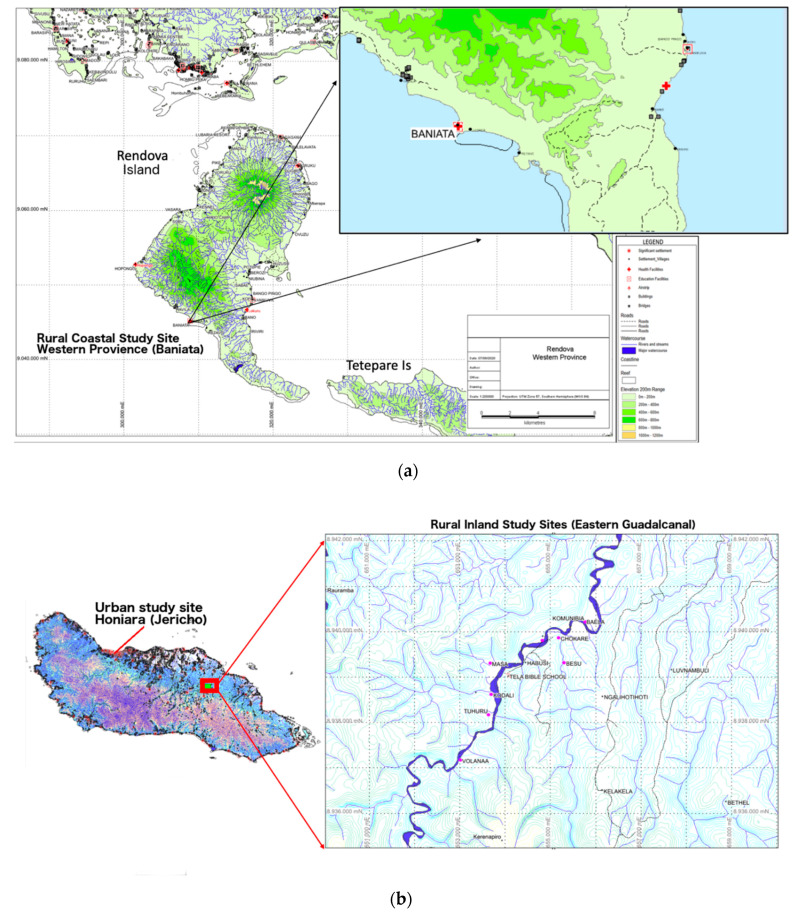
(**a**) Rural coastal study site, Baniata Village, Western Province. (**b**) Urban study site (Honiara, Jericho 1 and 2), and rural inland study sites (Eastern Guadalcanal). (**a**,**b**) are provided by Carlos Maelaua, a consulting geologist in the Solomon Islands.

This research took place in 3 geographically distinct sites across the Solomon Islands, including representation from both rural and urban populations. Snowball sampling was used at each of the 3 sites to ensure randomized sampling. The 2 rural study sites are distinguished by their access to the ocean. The first study site and subsequent 2 study sites were assessed 1 year apart during the same season. The first study site was Baniata village (visited August 2018), a rural coastal village comprised of ≈80 households (≈645 people) and surrounded by native bush and mountains (Figure 1a). Baniata is located on Rendova Island, Western Province, and is accessible only by boat from the neighboring region of Munda. The second study site (visited in 2019) comprised a collection of 8 smaller rural inland villages in Eastern Central Guadalcanal (Figure 1b; Besu, Chokare, Komunibia, Sili, Kukudu, Masa, Kodali, and Tughuru). Accessing these inland villages required a 3-h drive on dirt roads, followed by a 2-h hike through native bush. Travelling between villages required a local guide who led our team through the jungle and across rivers to reach individual households. Each village contained 5–10 households, with a total of ≈430 individuals and 50 households across all 8 villages. These rural inland villages were geographically and culturally similar enough to group them into one population. Rural coastal and inland sites had no cellular service, internet, or electricity (with the exception of a few small solar panels). The final study site was an urban community (visited in September 2019) located in the neighboring villages of Jericho 1 and Jericho 2 with ≈5000 people (Figure 1b). These urban populations serve as proxies for the larger urban population living in Honiara, the largest city and capital of Solomon Islands, with a total population of ≈84,520. Over 80% of Solomon Islanders live outside of Honiara in rural areas.

### 2.2. Study Design

We used an observational mixed-method cross-sectional study design. Quantitative surveys were administered to the woman in each participating household who was primarily responsible for household food preparation and agri-food cultivation. Dietary intakes were assessed using the repeat 24 h multiple-pass recall (MPR) method; questionnaires were used to assess knowledge, attitudes and practices (KAP), and food insecurity; and anthropometric measurements were undertaken. Qualitative key informant interviews were held in each village with village elders, and sometimes additional community members, in order to build understanding around current food systems and forecasted food system changes. 

The Solomon Islands is home to over 75 distinct languages. Research tools were translated and conducted in the most commonly used language, called pidgin, in order to accommodate language diversity. All survey materials and qualitative key informant questions were pretested in consultation with the nutrition and dietetics professionals from Solomon Island National University for cultural appropriateness, comprehension, and respect for indigenous perspectives and values. Prior to data collection at each study site, dietitian enumerators consulted with village chiefs to ensure sensitive topics were respected and activities that may be considered taboo were avoided.

The first phase of research took place in August 2018 in the rural coastal village of Baniata. The second phase, which included the inland rural sites in Eastern Central Guadalcanal and the urban site in Honiara, took place in September 2019. The second phase study design was modified slightly on the basis of adaptations and feedback from the first phase. 

Eight local dietitians/nutritionists from the Solomon Islands National University led the quantitative nutrition survey assessments and qualitative focus group discussions at each study site, and also attended a multiday training to ensure comprehension of the research methodologies. Ethical approval was obtained from the Massey University Human Ethics Committee (#4000020954), as was research clearance from the Solomon Islands Ministry of Education and Human Resources and Development (MEHRD #0668216) and the Solomon Islands National Health and Research Committee prior to commencing this study. Village chiefs were notified, and they granted consent before study commencement. 

### 2.3. Quantitative Nutrition Surveys

Households within each population were randomly selected using a snowball sampling technique. Nutritionists collected quantitative data from women (aged 15–49) who were primarily responsible for cultivating and preparing household foods (*n* = 94). Women were excluded if they were pregnant or lactating, due to increased energy and nutrient requirements [14]. Nutritionists walked to each selected household and conducted anthropometric measurements, 24 h multiple-pass food recalls (MPRs), and a nutrition survey in each participant’s kitchen (when appropriate). 

### 2.4. Anthropometry

Anthropometric measurements included height, weight, and body fat percentage for participants in all of the villages across the 3 study sites. Participants who lived in inland and urban populations were also measured for waist (within 0.1 cm) and calf circumference (within 0.1 cm). Height (within 0.1 cm) was assessed using a tape measure, and each nutritionist was trained accordingly with best practices, including the removal of excess clothing and having the individual stand on a flat surface without shoes [15]. Weight (0.1 kg) was measured using a portable digital weight scale (GreaterGoods Digital Body Fat Scale, Model 0391). Body fat percentages (0.1%) were assessed using a comprehensive bioelectric impedance body composition analyzer (InBody S10). Body mass index (BMI) was calculated from the participants’ height and weight (BMI = kg/m^2^). 

### 2.5. 24 H Multiple Pass Recalls

Quantitative 24 h multiple-pass recalls (24 h MPR) were conducted across 2 nonconsecutive days to represent realistic dietary intakes of participating women [16]. Dietary recalls were adapted to capture both species- and variety-level biodiversity of each consumed food. In addition to types and quantities of foods consumed, cooking methods and brands were also recorded. The sourcing of each ingredient was also captured, and included self-cultivated foods, wild foods, store-bought foods, market purchases, and takeaway foods. If the participant had leftover food or drinks from the previous day’s meal, nutritionists directly measured the amount consumed as determined by the participant (≈35% of meals). Portion sizes were estimated by the participant and weighed using digital kitchen scales (Etekcity model EK6015) or measured using graduated cylinders (500 mL and 1000 mL). If the food or drink were not available for direct measurement, then participants estimated quantities of food or drink using water, modelling clay, or strips of paper in the participant’s original dishware. Displacement techniques were utilized to determine portion sizes if clay or paper were used to determine portion size. After, food quantities were determined by converting the quantities or densities of the clay/paper by using food density conversion factor estimates from the FAO International Network of Food Data Systems (INFOODS) Density Database [17]. The interview also probed for ingestion of dietary supplements and alcohol. 

Food and ingredient source categories included self-cultivated or produced, wild collected, wet market, convenience store, or takeaway meals. Self-cultivated foods are foods intentionally produced by the household for consumption. Wild-collected foods included foods not intentionally cultivated but collected from forests, rivers, or the ocean. Wet market foods are locally sourced from a produce or meat market. Convenience store foods are those foods purchased within a brick-and-mortar shop or a canteen. Takeaway meals are ready-to-eat foods purchased from a street vendor or restaurant. 

Nutritional composition data and food groups were sourced from the Pacific Island Food Composition Database (Version 2) [18], Australia and New Zealand food composition databases, and the FAO/INFOODS databases [17]. Food Works (Xyris Version Version 10.0.1) was used to calculate nutrient losses and retentions from food preparation styles (i.e., boiling, drying, etc.). Food varieties that could not be identified in food composition databases were substituted for closely comparable foods. Usual nutrient intakes were calculated from 2 nonconsecutive day 24 h MPRs using the multiple source method (MSM) [19]. Diet consumption data were categorized into the food groups used in the Minimum Dietary Diversity for Women (MDD-W). The level of food processing was classified using NOVA 1–4 categories, with NOVA 4 capturing exclusively ultra-processed foods [20]. 

The Minimum Dietary Diversity Score for Women (MDD-W) is a proxy for the probability of micronutrient adequacy for women aged 15–49 [21]. Food groups were extracted from consumption data in 24 h MPRs and served as a binary indicator of dietary diversity. Diets that contain five or more food groups (out of a possible 10) have a higher likelihood of achieving micronutrient adequacy [21]. Dietary species richness (DSR) was used to assess agrobiodiversity, and was calculated by counting the unique number of species consumed during each 24 h MPRs [22].

### 2.6. FAO’s Food Insecurity Experience Scale (FIES)

The Household Food Insecurity Experience Scale (FIES) of the Food and Agriculture Organization of the United Nations [23] is an 8-item binary question scale designed to estimate annual household levels of food insecurity. Scores range from 1 to 8, with an average score from 1 to 3 classified as low annual household food insecurity, 4 to 6 as moderate, and 7 to 8 as severe [24]. The FIES is an indicator for SDG 2 (2.1.2). 

### 2.7. International Physical Activity Questionnaire (IPAQ-SF)

The International Physical Activity Questionnaire Short Form (IPAQ-SF) is a 7-item survey designed to quantify weekly physical activity and related intensities for each participant. The IPAQ-SF was adapted to accommodate common activities throughout the Solomon Islands. Three categories of physical activity were used for comparison: low activity, moderate activity, and high activity. Results were converted into MET minutes (metabolic equivalent of task). IPAQ-SF was calculated and analyzed using the IPAQ scoring protocol outlined by the IPAQ Group [25], classified as low, moderate, or high activity. IPAQ-SF was added to the surveys for the second phase of the study in rural inland and urban sites but were not included in the rural coastal site.

### 2.8. Knowledge, Attitudes, and Practices (KAP)

Following FAO guidelines, we asked a series of questions to assess the participants’ knowledge, attitudes, and practices (KAP) regarding nutrition, agricultural practices, and food waste [26]. The KAP questions were designed to identify specific barriers to accessing and preparing healthy foods. All KAP questions were pretested with local dietitians to ensure comprehension and cultural sensitivities. Data were analyzed to identify how women’s knowledge and attitudes influenced practices related to household food preparation. Post-harvest garden and household food waste was estimated by the primary cook of household, with primary foods and reasons for loss recorded.

### 2.9. Quantitative Data Analysis

Nutrition surveys including 24 h MPRs, KAP, anthropometric measurements, and descriptive data were analyzed using IBM SPSS (Version 25), Tableau (Version 2020.1), RStudio (1.2.5001), and Xyris FoodWorks (Version 10.0.1). 

Percentage of participants consuming less than the estimated average requirement (EAR) was used to estimate the prevalence of inadequate nutrient intakes. EAR is defined as the average daily nutrient intake level that is estimated to meet the requirements of 50% of the healthy individuals in a particular life stage and gender group [27]. The population prevalence of inadequate intakes was computed using the EAR cut point method for each unique study site [28]. The EAR cut point method was computed by calculating the proportion of individuals with usual intakes below the EAR for calcium, vitamin B_12_, folate, selenium, potassium, vitamin A_eq_, thiamine, and zinc. The full probability approach was used to determine average probability of inadequacy for iron. This approach is necessary to adjust for absorption limits and iron losses among menstruating women [(Observed Intake × Upper limit) − 0.87(assumed basal loss of iron)] [29].

The World Health Organization and the FAO recommend a minimum of 400 g of non-starchy fruit and vegetables (NSFV) per day to prevent chronic diseases such as heart disease, cancer, diabetes, and obesity, as well as to prevent and alleviate several micronutrient deficiencies, especially in less developed countries [30]. NSFV intakes were extracted from 24 h MPRs and compared to the WHO/FAO recommendation of 400 g/day. 

Linear regression models were used to demonstrate the relationship between body fat percentage and the average number of species consumed (DSR), knowledge of healthy diets, and consumption of dark leafy vegetables, with *p* ≤ 0.05 regarded as significant.

### 2.10. Estimation of Misreporting of Dietary Intake Data

Misreporting of dietary intake data was controlled for using Goldberg cutoff points. Cutoff points were calculated by comparing energy intakes (EI) with estimated basal metabolic rate (BMR_est_) using the Harris–Benedict equation [BMR = (10 × Weight) + (6.25 × Height) − (5 × Age) − 161] [31]. Underreporting was defined as EI: BMR_est_ < 1.15, and overreporting as > 1.96 [32].

### 2.11. Village Comparisons

A one-way ANOVA was used to determine intra-village differences in anthropometric, lifestyle, and diet quality data. Normality checks and Levene’s test were conducted and the assumptions met. Post hoc comparisons using the Tukey test were carried out. 

### 2.12. Qualitative Analysis

Key informant interview questions were structured on the basis of previously conducted surveys in the Solomon Islands aimed at characterizing the sustainability of indigenous food systems. Data were summarized to contextualize quantitative findings using the qualitative software NVIVO 12 (Version 12.6).

## 3. Results

### 3.1. Food System Comparisons

Comparisons between three geographically distinct indigenous Solomon Island food systems are provided for rural coastal, rural inland, and urban populations in Table 1. Clear distinctions were found between rural and urban populations, including population size, accessibility, proximity to markets, agri-food production, and wild food collection. 

### 3.2. Excluded Data

One participant’s dietary data were removed due to underreporting (Goldberg cutoff point of >1.96). 

### 3.3. Anthropometric and Physical Activity Measures

Rural populations, on average, had significantly lower body fat percentage, BMI, and waist circumference when compared to urban populations (Table 2). Rural villagers exerted 853 more MET minutes per week of physical activity than the urban population, with the majority of rural participants achieving “high activity” levels. However, 90.1% of urban and 93.8% of inland rural participants achieved the physical activity recommendations of 600 weekly MET minutes set by the WHO. BMI was highly correlated with both body fat percentage (*p* ≤ 0.0001, *r*^2^ = 0.73), waist circumference (*p* ≤ 0.0001, *r*^2^ = 0.77), and calf circumference (*p* ≤ 0.0001, *r*^2^ = 0.47).

### 3.4. Dietary Quality and Diversity

Diets in rural villages (both coastal and inland) largely were predominately sourced from self-cultivated and wild foods, including root vegetables (taro, cassava, and kumara), bananas (cooking and eating), dark green leafy vegetables (kasume fern, slippery cabbage), and coconut products (cream). Both types of rural villages sourced significant protein from canned tuna, but differences existed in terms of access to wild food. Coastal villagers wild-collected protein from the ocean and the bush, whereas inland villagers sourced their protein from the nearby river (ora, grey fish, and wild pig) as well as from the bush. 

Diet quality differed significantly among the three study populations (Table 2). Dietary species richness (DSR) was significantly higher in rural populations. The number of species consumed was correlated to the utilization of wild and cultivated foods in both urban and rural populations (*r* = 0.13, *p* = 0.003). The DDS mean score for rural coastal populations was significantly higher than for the rural inland population. The average number of participants across three sites achieving >5 DDS was 17.2%, indicating a high percentage of participants are unlikely to achieve nutrient adequacy. The majority (>75%) of participants in rural areas achieved the WHO recommendation of >400 g of non-starchy fruits and vegetables daily, compared to less than half (42.2%) of the urban population who met this recommendation. Ultra-processed foods (NOVA 4) were consumed exponentially according to proximity to the urban center, and the most commonly consumed such foods were white breads, instant noodles, donuts, Milo drink mix, milk tea, and sausages.

### 3.5. Energy and Nutrient Intakes 

**Figure 2:**
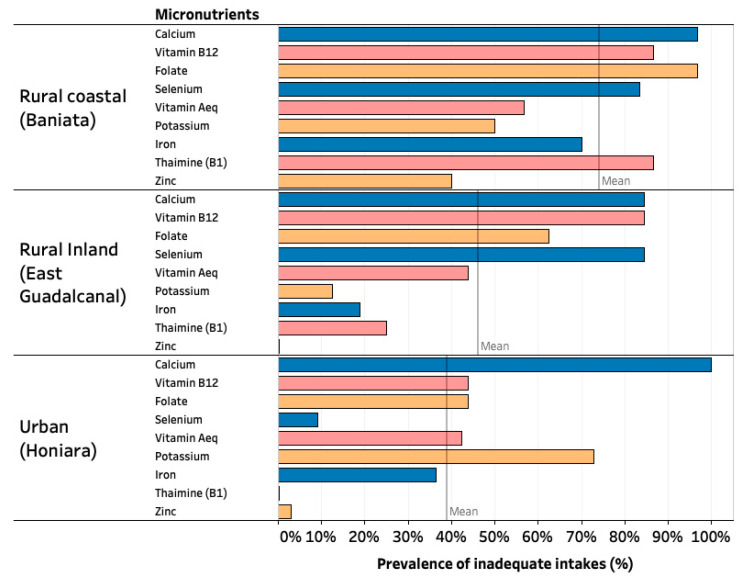
Prevalence of inadequate micronutrient intakes within three geographically distinct indigenous Solomon Islands food systems (*n* = 94).

Energy, macronutrient, and micronutrient intakes for the study populations are compared in Table 3. The prevalence of inadequate micronutrient intake of each population is indicated in Figure 2. The rural coastal village had the highest prevalence of participants who did not achieve the EAR for micronutrients of concern, followed by the rural inland and urban sites. More than half of the calcium intake for the rural populations came from dark green leafy vegetables, including slippery cabbage, wild fern, and leaves from root crops. Potassium was low for the urban population due to lower intakes of green leafy vegetables and roots, tubers, and bananas. The prevalence of inadequate vitamin A_eq_ and thiamine intakes were lower in the rural inland and urban food systems in part due to the rice fortification policy mandate in November 2018, which was enacted after data collection in the rural coastal food system. However, both rural food systems sourced significant quantities of vitamin A_eq_ from cultivated, purchased, or wild collected dark green leafy vegetables.

Nutrients that were low in rural food systems but not in urban ones were vitamin B_12_ and selenium. Urban food systems sourced the majority of their vitamin B_12_ from canned tuna (taiyo), and selenium was sourced from canned tuna (taiyo) as well as from fortified white rice. Low consumption of animal-sourced foods, including fish, dairy, and meats, contributed to low vitamin B_12_ intake in the rural villages. All three study sites, on average, did not exceed sodium recommendations.

**Figure 3:**
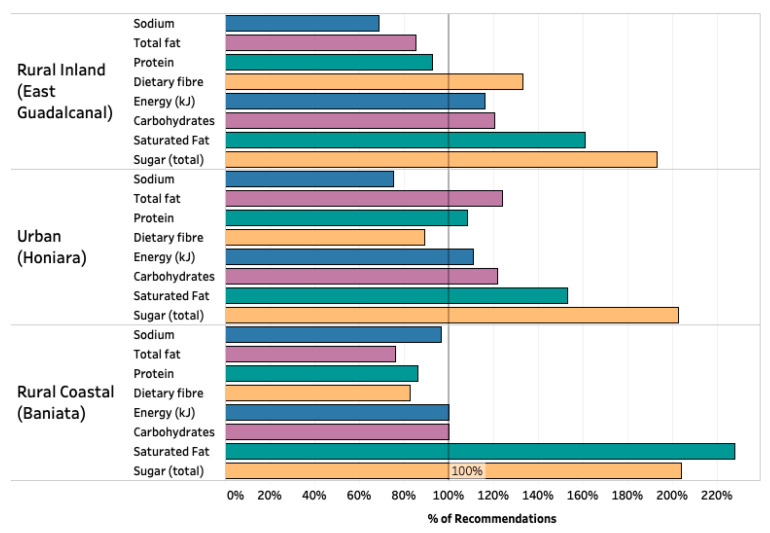
Macronutrient intakes compared with dietary recommendations in three geographically distinct food systems (*n* = 94). Energy requirements were compared to total energy expenditure (TEE) from basal metabolic rate (BMR) + physical activity level (PAL). Total fat recommendations <30% total kcal and saturated fat recommendations <10% of total kcal [33]. Sugar limits are sourced from WHO guidelines (2015) and salt limits from American Heart Association guidelines (2020).

Macronutrients in all three food systems supplied higher than the recommended intakes of saturated fats and total sugars (Figure 3). Saturated fats were primarily sourced from coconut and coconut products. Sugars were primarily included in the diet in the form of sugar-sweetened beverages. Only the rural inland food system was able to meet fiber recommendations. On average, rural populations did not achieve protein recommendations, whereas the urban population did (Figure 3). Overall, mean energy consumption for each population was met; however, deficiencies in essential nutrients remain, including protein, vitamin B_12_, and calcium. 

**Figure 4:**
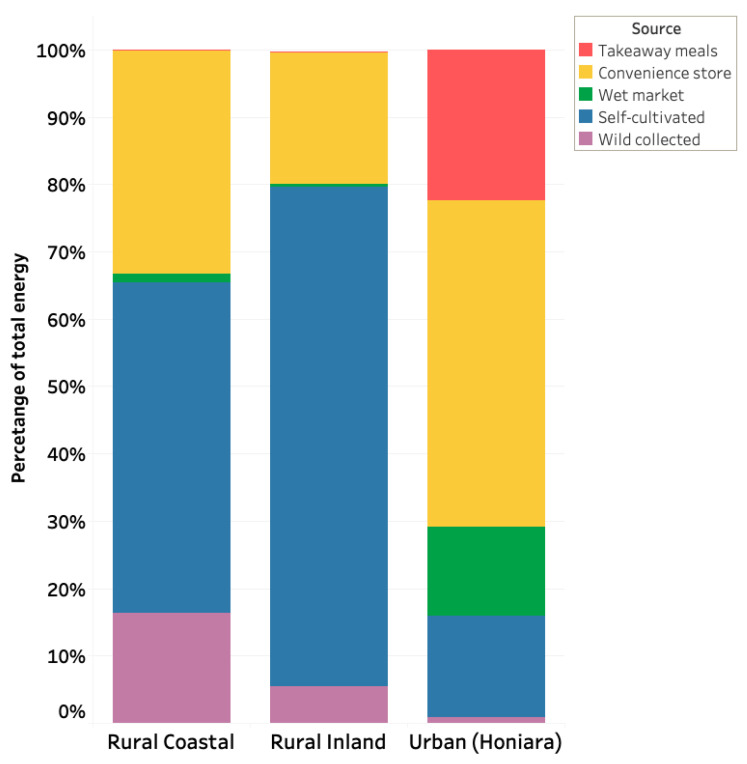
Energy food source patterns between three geographically unique indigenous Solomon Island populations (*n* = 94).

Using a linear trend model, we found a significant decline in dietary energy (kJ) sourced from cultivated and wild collected foods among younger participants when compared with older participants in both rural and urban settings (*p* ≤ 0.001). There was a significant increase of store-bought foods in the urban population compared to the rural populations (Figure 4). Energy sources from wild foods were negligible in the urban populations when compared to the rural populations.

Table 4 compares the top five food sources of energy, iron, calcium, vitamin A_eq_, and zinc in the rural inland and rural urban populations. Food energy from rural inland diets was sourced primarily from coconuts, root crops, and fortified white rice. In contrast, diets in the urban population were characterized by fortified white rice, fortified refined grain products, coconuts, cassava, and added sugars. Fortified white rice was the top contributor of zinc and iron for both rural inland and urban participants. 

Through a linear regression analysis, we found that participants who had a higher knowledge of healthy diet patterns, consumed a wider diversity of species (DSR), and consumed more dark green leafy vegetables (by weight) were significantly more likely to have healthier body fat percentages (*p* ≤ 0.001, *R*^2^ = 0.261). No significance was found between participants’ body fat percentage and total energy consumption from NOVA 4 ultra-processed foods.

**Figure 5:**
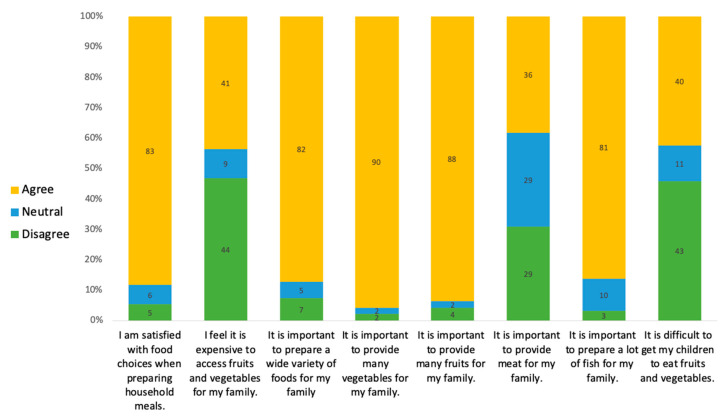
Knowledge, attitudes, and practice survey results among rural and urban women (*n* = 94).

Results from the KAP survey (Figure 5) indicated that most women felt it was important to provide fruits (93.6%) and vegetables (95.7%) for their families (*n* = 94). Rural population attitudes towards the affordability of access to fruits and vegetables were slightly but significantly more favorable than among urban participants. Nearly half (47.5%) of rural women felt meat was an important part of the diet, whereas only 21.7% of the urban population shared this view. There was a significant increase in urban women who felt it was difficult to get their children to eat fruits and vegetables when compared to the rural population (27.7% and 50.1%, respectively). 

### 3.6. Qualitative Key Informant Interviews

Village elders are the leaders of their respective communities in the Solomon Islands and are the primary host of the villager’s traditional knowledge. Results for qualitative key informant interviews from each village elder’s food system were summarized and categorized into three primary areas:

1. Traditional knowledge loss: Elders from all three study sites expressed their interest in sharing indigenous and traditional knowledge with the younger generations, but children were often unwilling to listen unless required. One elder summarized this concern by stating, “All traditional knowledge is passed, but kids do not want to do it.”
2. Traditional food declines: Seasonal fluctuations of market produce prices within the urban population impact the ability to purchase certain locally grown foods. One elder added, “However, rice has filled the void of these fluctuations and cost barriers,” and another added that “adults get tired of rice all the time, but kids only want rice. Now, the kids’ preferences are influencing parents’ preferences, too.” Rural village elders expressed that local breeds and varieties are decreasing overall. Rural inland populations now cultivate their agri-foods 1 h away (walking) from their village to be closer to the road for easier market access, but this now limits the quantity of traditional foods that are carried back to the village. Additionally, rural inland villagers and urban villagers on the island of Guadalcanal are facing the recent threat of invasive giant African land snails (*Achatina fulica*), which decimate crops by the thousands. One elder stated, “We used to plant slippery cabbage near our house, but now the snails eat them all.”
3. Climate change and weather patterns: Urban and rural elders expressed their concerns about climate change and associated weather pattern changes. Respondents said that dry seasons have decreased and that increases in rain throughout the year have caused many crops not to grow as well. One village elder shared the challenges to the local food system by stating, “We used to listen to the weather, but now we cannot.”

## 4. Discussion

We found substantial differences in anthropometric measures, macronutrient and micronutrient intakes, and MET minutes between rural and urban Solomon Island populations. We also found significant differences in food sourcing patterns between rural and urban populations, with urban populations often replacing wild or self-cultivated agri-foods with purchased and ultra-processed foods. Overall, participants in rural areas sourced more energy from wild and cultivated foods; consumed a wider diversity of foods; had a higher probability of meeting WHO recommendations of >400 g of NSFV daily; were more active physically; and had significantly lower body fat percentages, waist circumference, and BMI when compared to urban populations. Overall, an average of 17% of the study population achieved dietary diversity scores (MDD-W) ≥5, slightly higher than recent diet diversity findings from populations living in the semi-urban areas of Malaita [13].

Since our data collection in the rural coastal village of Baniata in 2018, rice, wheat flour, and vegetable oil have been fortified with zinc, iron, vitamin A_eq_, and thiamine (2019). Since a larger proportion of participants achieved intakes above the EAR for these micronutrients in both rural inland and urban populations, fortification may be a significant influencing factor of nutrient adequacy for these select nutrients. Fortified white rice was the main source of zinc for rural inland and urban populations. Salt is fortified with iodine, and most participants achieved the EAR. Food fortification may solve single micronutrient deficiencies but could ultimately reduce the sustainability of indigenous food system and give rise to diet-related noncommunicable diseases (NCDs), particularly since most fortified foods are imported and tend to be processed.

Urban populations consumed significantly more protein and ultra-processed foods (NOVA 4), were more likely to eat takeout foods, and had less diverse diets compared to rural populations. Less than half of urban participants met their recommended NSFV intakes. Whole, minimally processed foods contain a wide diversity of antioxidants, phytochemicals that protect against heart disease, T2DM, and obesity [34,35,36], of which urban populations are less likely to eat due to lack of accessibility. If ultra-processed foods (NOVA 4) continue to displace traditional foods, as evidenced by trends across the broader Pacific [6], NCD risks will likely continue to rise, even if levels of essential micronutrients are met through fortified foods. Additionally, modelling of staple crops predict that protein content and micronutrients of rice and wheat will decline significantly as atmospheric carbon dioxide rises [37]. Populations that rely heavily on single staple foods as their primary source of nutrition will experience proportional adverse impacts of these nutrient losses, aggravating existing cases of malnutrition and encouraging new ones [38].

Seafood is a critical and culturally important food for achieving food and nutrition security in the Solomon Islands. Urban participants sourced vitamin B_12_ primarily from canned tuna (taiyo), as well as selenium from canned tuna and fortified white rice. Low consumption of animal-sourced foods, including fish, dairy, and meats, contribute to low vitamin B_12_ and protein intake in rural populations. Low vitamin B_12_ intake can result in irreversible neurological damage if B_12_ is underconsumed for long periods [39]. Some options to ensure adequate vitamin B_12_ intake within vulnerable populations include the promotion of more animal-sourced foods, supplementation, and fortification. Tuna catches are predicted to rise in PSIDS over the next 50 to 80 years due to changing ocean currents, potentially serving as a regionally abundant source of protein and other essential nutrients for current and future food systems [40]. In the short term, canned fish can help fill the gap between sustainable coastal fish production and recommended fish intakes [40].

Dietary diversity was found to be lower in the rural inland site than in the rural coastal site, which is likely related to the inland villagers’ current struggle with the invasive giant African land snail (*Achatina fulica*). This destructive snail appeared in Guadalcanal rivers nearly three years ago, and has caused widespread damage to numerous crops of value, including kumara, slippery cabbage, and bananas. Villagers shared that their tolerance is low for this invasive species and that it is causing great stress. More data are needed examining the impacts from African snails in relation to diet quality, particularly for maternal and child health. While snails are not currently consumed, they are edible (when properly prepared), and provide essential trace minerals needed for optimal growth and development, including iron, magnesium, calcium, phosphorus, and potassium [41]. If culturally appropriate, education around the preparation and consumption of giant African snails may help to mitigate malnutrition in vulnerable populations such as children and pregnant women. An additional contribution to reduced dietary diversity within the rural inland site is that home gardens have recently moved further away from homes (a 1.5 h walk) and closer to the main road where transportation is available to reach produce markets. This increased distance from gardens to homes may impact household diet diversity as the food’s weight and space are of concern when hiking back from the gardens.

Calcium is a nutrient of concern for all populations. Calcium-rich foods are primarily sourced from starchy staples and dark green vegetables, with the exception of the urban population, who source most calcium from refined grain products. Betel nuts (*Areca catechu*) are a commonly chewed stimulant drug, used regularly by 45% of participants in urban and rural sites. Consumed alongside the nut is dried, crushed coral (calcium carbonate; CaCO_3_), which may provide >100% of the user’s EAR for calcium (3 g = 1000 mg, or >100% EAR). However, betel nuts are a highly addictive and accessible cancer-causing drug [42].

Ultra-processed foods (NOVA 4) have the worst nutrient profiles yet are becoming the most prevalent foods within global food systems, including in the neighboring countries of Australia and New Zealand [43,44]. NOVA 4 were consumed in all three study sites, with the highest consumption within the urban population. Interestingly, the consumption of ultra-processed foods had no correlation with body fat percentage or BMI. However, numerous global studies have found significant inverse associations between consumption of NOVA 4 foods and fiber, potassium, and micronutrients [45]. The most common ultra-processed foods were doughnuts, bread, instant noodles, Milo drink mix, and sausages. Takeaway meals consisting of fried meats or fish, sausages, and rice were more common in the urban populations. Fewer ultra-processed foods have made their way to rural areas, likely due to the journey required to access the villages.

Our findings suggest that higher body fat percentages were highly correlated with higher BMIs and waist circumferences, and therefore body fat percentage was used as a primary health indicator due to its relation to NCDs and chronic disease risk [46,47,48]. We found that lower body fat percentages were significantly correlated with a greater intake of unique numbers of species, higher knowledge of healthy diets, and increased intakes of dark leafy green vegetables. These results align with observations from Tsuchiya et al. (2017), where lower frequencies of green leafy vegetable consumption and dietary diversity were associated with increased rates of obesity in the urban setting of Honiara [49].

Food insecurity was classified as moderate in the rural coastal community and classified as low in the rural inland and urban communities. Food security is often provided through “insurance crops”, such as swamp taro; kasume (wild fern); and, more recently, imported white rice. Nutrition security, however, is nonetheless a cause for concern, as adequate supplies of essential nutrients are not available year-round per current food system availability [50].

Recent findings from Solomon Island populations indicate that overall nutrition knowledge is weak, which can impede informed choices regarding food consumption [13]. Additionally, urban women in our study perceived getting children to eat fruits and vegetables to be twice as challenging as women in the rural settings. This difference could be related to changes in urban food environments, including advertisements, lack of cultivation opportunities, or perceptions that traditional foods are old-fashioned. Nearly half of rural women felt meat was an important part of the diet, whereas only 21.7% of urban participants shared this view. This could be related to the sourcing of meat, in that in rural settings meat is typically sourced from the bush, whereas in urban settings meat is typically consumed via less healthy sources such as processed sausages or fried chicken.

Both urban and rural village elders expressed their concerns regarding the loss of traditional knowledge, as well as concerns for an increasing reliance on less healthful, imported foods. While imported foods can fit into a healthful diet, it is important to recognize the potential sustainability trade-offs associated with displacing traditional foods rich in nutrients with energy-dense imported and ultra-proceeded foods. Other Melanesian PSIDS, such as Fiji, have much higher rates of childhood and adult obesity compared to the Solomon Islands, likely associated with a lengthier exposure to energy-dense and nutrient-poor foods [51].

### 4.1. Implications and Further Research

Indigenous knowledge can help build local food system resilience, strengthen food and nutrition security, and help to inform the global debate on improving the sustainability of global food systems [52]. Studies have identified that agroecological approaches informed by participatory research and indigenous knowledge can help empower communities and increase food sovereignty [53]. Neglected and underutilized agri-food species also have the potential to generate income for farmers, meet demand in local markets, and contribute to meeting UN sustainability goals [1].

A recent technical report identified community food production initiatives in PSIDS as part of the solution for addressing food and nutrition insecurity by increasing dietary diversity and incomes while reducing household food expenditure [54]. Strategies aiming to improve the nutrition and health outcomes of indigenous food systems should begin with the inclusion of traditional knowledge, values, and priorities. Additionally, convenience, affordability, and income-generating opportunities are important considerations when aiming to improve food systems’ contributions to human and planetary health. Future research should examine how the multiple dimensions of sustainable diets, including nutritional, environmental, economic, and sociocultural, can be achieved. Utilizing and promoting the food-based dietary guidelines for the Solomon Islands can help guide policies and educational efforts towards culturally significant, nutritious, and balanced diets.

Lastly, a deeper examination of ingredients used within ultra-processed foods is warranted, particularly those containing trans fatty acids under the ingredient name “partially hydrogenated oil”. Trans fatty acids have historically been poorly displayed on nutrition facts labels in Solomon Islands, findings that are confirmed by a large-scale study examining completeness of nutrition information facts with 6000+ food and drinks in Fiji [55]. Trans fatty acids, even in small quantities, are extremely deleterious to cardiovascular health and overall NCD risk [56].

### 4.2. Limitations

The 24 h multiple pass dietary recall methodology has not been adapted for a Solomon Islands population, and therefore population-specific adjustments are not known. However, we countered this limitation by using the Goldberg cutoff methodology to reduce the likelihood of under- and over-reporting. Another limitation is that BMI cut points have not been established for Melanesian populations, and therefore we used additional metrics such as body fat percentage and waist circumference. There was a one-year span between the first round of data collection (2018) and the second (2019). Researchers controlled for this by conducting the study during the same time of year, but also acknowledge this as a limitation. Sample populations were selected to represent three geographically distinct environments in which the majority of Solomon Islanders live. However, Solomon Islands is an archipelago of 900+ islands with over 75 distinct languages, and therefore each community has unique challenges and opportunities when aiming to obtain food and nutrition security. Additional food systems research is required to further understand dietary diversity, quality, and transitions in more remote island locations. Food composition data are severely lacking for a wide diversity of available varieties of foods across the Pacific. Updating the Pacific food composition tables can help provide food-based and culturally significant solutions to mitigating malnutrition.

## 5. Conclusions

Clear anthropometric, diet-quality, and sourcing differences were found between rural and urban participants. We found urban populations to be at a significantly increased risk for obesity and NCDs. Estimated requirements of zinc, iron, folate, and vitamin A_eq_ were met by the majority of participants after fortification mandates for rice, flour, and oil were enacted in November 2018. These fortifications should improve malnutrition outcomes for vulnerable populations. However, fortified foods may artificially inflate individuals’ confidence in the quality of their diets, since our findings indicate that traditional foods are being displaced by imported and ultra-processed foods. As urbanization increases, declines in knowledge of traditional agri-food are accelerated through shifts towards industrially processed foods and changes in the taste preferences of younger generations.

Villagers expressed strong interest in understanding how they can improve their diets to achieve better nutrition outcomes within their communities. Elders expressed grave forecasts about traditional knowledge losses, changing dietary patterns, and climate change. Certain processed foods, particularly those which are locally produced, can play a critical role in achieving food and nutrition security as well as food sovereignty—a critically important concept given the recent food system disruptions caused by the COVID-19 pandemic. Unless action is taken to preserve and integrate traditional knowledge, associated food and nutrition security benefits will likely continue to rapidly erode. There are clear opportunities to leverage traditional knowledge and improve cultivation and consumption of neglected and underutilized species that can help build more sustainable and resilient food systems while ensuring indigenous knowledge and cultural preferences are preserved.

## Figures and Tables

**Table 1:** Characteristics and food system descriptors for three geographically unique indigenous Solomon Island populations.

Descriptor	Rural Coastal	Rural Inland	Urban
Sample size	Households (*n* = 30)	Households (*n* = 32)	Households (*n* = 33)
Village name (s)	Baniata	Multiple villages (Besu, Chokare, Komunibia, Sili, Kukudu, Masa, Kodali, Tughuru)	Jericho 1 and Jericho 2
Location	Coastal village on Rendova Island in the Western Province	Eastern rural inland villages, Guadalcanal	Honiara (capital city), Guadalcanal
Population	≈645 villagers and 80 households	≈430 villagers and 50 households	>84,500 (total population)
Season	Lean season (July/August 2018)	Rainy season (August/September 2019)	Rainy season (August/September 2019)
Food insecurity (FIES)	FIES Composite: 4.1 Moderate food insecurity	FIES Composite: 2.5 Low food insecurity	FIES Composite: 2.2 Low food insecurity
Household monthly income (average)	SBD 1043 (USD 125) [SD SBD 416 (USD 53)]	SBD 965 (USD 115) [SD SBD 569 (USD 68)]	SBD 1115 (USD 133) [SD SBD 719 (USD 86)]
Household size (average)	6.5 people per household (SBD 160 pp/month)	5.1 people per household (SBD 193 pp/month)	6.9 people per household (SBD 161 pp/month)
Accessibility	Village access requires a 90-min commute from the regional capital of Munda on a wooden petrol-powered fishing boat.	Village access requires a 3-h drive on dirt roads and across rivers from the capital city of Honiara, followed by a 2-h trek to reach inland river villages.	Villages are centrally located within the urban capital of Honiara.
Proximity to external markets	The closest wet market was located in Noro, which requires boat access. Baniata had 2 boats, which limits the number of villagers who are able to sell their agri-food products each day.	The closest wet market is in Honiara (above) and takes a considerable amount of time to access.	Neighborhood markets external to Jericho exist, but the Honiara central market is the closest. Walking would take 1.5 h, and a bus would take 30 min (during business hours).
Internal markets or canteens	An internal canteen exists with a limited selection of basics such as noodles, flour, oil, rice, biscuits, candies, and tobacco products.	An internal canteen exists with a limited selection of basics such as noodles, flour, oil, rice, biscuits, candies, and tobacco products.	No internal market exists, but street foods, select produce, and basics are available for sale directly outside of the village.
Agri-food cultivation and production	All women participated in agri-food cultivation and production and market sales.	All women participated in agri-food cultivation and production and market sales.	Women were less involved with agri-food production, cultivation, and sales and had a wider variety of responsibilities, including the formal sector or as a caretaker.
Wild food access	Ocean and bush were accessible to all villagers, and wild foods were collected to supplement diets.	River and bush access were accessible to all villagers, and wild foods were collected to supplement diets.	Ocean and bush access were not accessible to villagers, and wild foods did not play a large role in dietary intakes.
Food loss and waste, and preservation	A total of 26.9% (SD 16.5) of food was self-reported lost or wasted, with primary foods being vegetables, starchy staples, and nuts/seeds; ≈30% of villagers dried or smoked food for preservation.	A total of 29.1% (SD 11.3) of food was self-reported lost or wasted, with primary foods being vegetables, fruits, and starchy staples; ≈25% of villagers dried or smoked food for preservation.	A total of 31.1% (SD 11.1) of food was self-reported lost or wasted, with primary foods being vegetables, fruits, and starchy staples. Few in the urban setting practiced food preservation techniques.

**Table 2:** Mean anthropometric, health, and diet quality indicators across rural coastal, rural inland, and urban Solomon Island populations (*n* = 94).

Indicator	Rural (Coastal)	Rural (Inland)	Urban	Overall (Average)
Anthropometrics and health				
Age	37.1	39.8	37.0	37.9
Body fat percentage (%)	30.1	30.6	35.9 *	32.4
BMI	26.1	26.7	30.2 *	27.7
Waist circumference (cm) *	-	90.9	96.8 *	93.9
Calf circumference (cm) *	-	35.4	37.6 **	36.5
MET minutes (average/week) *	-	4338 *	3503.2	3920.6
% Low activity	-	6%	13%	9.5%
% Moderate activity	-	41%	54%	47.5%
% High activity	-	53%	33%	43%
Diet quality				
Dietary species richness (DSR)	7.1 *	6.7	5.8	6.5
MDD-W (DDS)	4.2 **	3.8	3.7	3.9
% DDS ≥ 5	26.6% **	13.1%	12.1%	17.2%
% >400 g NSFV	79.2%	77.4%	42.2% **	66.2%
Diet % ultra-processed (NOVA 4)	6.8	11.7	17.9 *	12.13
Takeout (#/week) *	-	0.3	1.3 *	0.8

* *p* ≤ 0.001, ** *p* ≤ 0.01. Waist circumference, calf circumference, MET minutes, and takeout data not available for rural coastal village.

**Table 3:** ANOVA comparisons of usual macro- and micronutrient intakes among three unique populations (*n* = 94).

Nutrient	Rural Coastal	Rural Inland	Urban	Overall Average
Macronutrients				
Usual energy intake (kJ)	7648.3	8549.7	9067.7 **	8421.9
Calories (kcal)	1828.0	2043.4	2167.2 **	2012.9
Total fat (g)	62.1	79.7 **	63.7	68.5
*Saturated fat (g)*	52.5 *	43.8	33.9	43.4
Carbohydrates (g)	224.7 *	308.2	328.6	287.2
*Sugars (g)*	61.7	57.9	60.7	60.1
*Dietary Fiber (g)*	22.8	33.2 *	20.6	25.5
Protein (g)	40.7	42.2	56.5 *	46.5
Micronutrients				
Vitamin A eq (µg)	379.8	908.9 *	599.7	629.5
Vitamin B_1_ (mg)	0.67	1.5 *	2.2 *	1.5
Vitamin B_2_	0.61	0.81	0.64	0.69
Vitamin C (mg)	84.7	193.4 *	111.7	129.9
Calcium [Ca] (mg)	290.7	483.7 *	320.9	365.1
Sodium [Na] (mg)	1376.8	1506.2	1934.2 *	1605.7
Potassium [K] (mg)	3204.6 *	4386.5 *	2284.9	3292.0
Magnesium [Mg] (mg)	416.8	504.8 *	257.6	393.1
Iron [Fe] (mg)	11.4 *	16.9	16.1	14.8
Zinc [Zn] (mg)	8.17 *	14.6	16.7	13.2

* p ≤ 0.001, ** *p* ≤ 0.01.

**Table 4:** Top five species contributing to energy, iron, calcium, vitamin A_eq_, and zinc in diets.

#	Rural Inland	% Total	Urban	% Total
Energy (kJ)				
1	Coconuts	24.07%	White rice	28.13%
2	Bananas	21.26%	Refined wheat products	18.36%
3	White rice	14.87%	Coconuts	12.64%
4	Taro (roots, leaves)	7.03%	Cassava	10.01%
5	Sweet potatoes	4.73%	Sugars (added)	6.54%
Iron				
1	White rice	21.11%	White rice	44.37%
2	Taro (roots, leaves)	13.27%	Slippery cabbage (bele)	9.17%
3	Fern (wild)	11.20%	Refined wheat products	14.86%
4	Coconut	14.11%	Cassava (roots, leaves)	6.58%
5	Slippery cabbage (bele)	7.41%	Tuna (canned, fresh)	1.77%
	*% of women below EAR*	18.8%	*% of women below EAR*	36.4%
Calcium				
1	Taro	38.68%	Refined wheat products	27.57%
2	Slippery cabbage (bele)	17.00%	Slippery cabbage (bele)	27.36%
3	Fern (wild)	2.73%	Cassava (roots, leaves)	9.00%
4	Pumpkin	8.22%	Coconuts	2.76%
5	Sweet potato	4.41%	Tuna (canned, fresh)	1.10%
	*% of women below EAR*	84.4%	*% of women below EAR*	100.0%
Vitamin A (eq)				
1	Sweet potato	71.21%	Slippery cabbage (bele)	32.95%
2	Slippery cabbage (bele)	19.93%	Cassava (roots, leaves)	26.20%
3	Fern (wild)	13.88%	Pumpkin (fruit, leaves)	14.03%
4	Taro (roots, leaves)	11.01%	Oil (fortified)	4.58%
5	Pumpkin (fruit, leaves)	7.33%	Taro (roots, leaves)	2.62%
	*% of women below EAR*	43.8%	*% of women below EAR*	42.4%
Zinc				
1	White rice	29.94%	White rice	53.45%
2	Taro (roots, leaves)	16.85%	Cassava (roots, leaves)	21.79%
3	Cassava (roots, leaves)	11.15%	Refined wheat products	4.32%
4	Fern (wild)	9.33%	Coconuts	3.17%
5	Coconuts	8.85%	Slippery cabbage (bele)	1.75%
	*% of women below EAR*	0.0%	*% of women below EAR*	3.0%

## Data Availability

The data presented in this study are available freely upon request from the corresponding author.
